# Supplementary material for: Relation of Dietary Patterns and Nutritional Profile to Hepatic Fibrosis in a Sample of Lebanese Non-Alcoholic Fatty Liver Disease Patients
Source: Nutrients. 2022 Jun 20;14(12):2554. doi: 10.3390/nu14122554 (PMC9229197; doi:10.3390/nu14122554)
Supplement: Supplementary file 1 [file nutrients-14-02554-s001.zip › File S1.pdf]

Supplemental File 1

Questionnaire of the study: ***Relation of dietary patterns and nutritional profile to hepatic fibrosis in a sample of Lebanese NAFLD patients***

Date dd/mm/yy

These questions are designed to be filled or to be circled according to the appropriate answer

Identification number………. **Phone Number…………….**

1. **Socio-demographic variables**
   1) Nationality………
   2) Place of residency………...
   3) Place of birth…...
   4) Age: ……….
   5) Gender: 1. Female 2. Male
   6) Marital Status: 1. Never married 2. Married 3. Divorced 4. Widowed
   7) Since how many years (widowed)? ……………
   8) Number of children: 1. More than 4 2. 2 - 4 3. Less than 2 4. No children
   9) Number of co-residents by room: ……
   10) Occupation: 1. Self-employed 2. Employed 3. Unemployed 4. Retired 5. others
   11) Education: 1. Illiterate 2. Primary 3. Secondary 4.University

2. **Anthropometric measurements**
   1) Height (cm)……..…
   2) Weight (Kg)……….
   3) BMI (Kg/m^2) ………
   4) Waist circumference (cm)……….
   5) Waist circumference /Hip circumference (cm)………

3. **Arterial pressure** (mm Hg) - Systolic/Diastolic:

4. **Blood tests (fasting state)**
   1) Cholesterol (mmol/L)
   2) LDL-Ct (mmol/L)
   3) HDL-Ct (mmol/L)
   4) Triglycerides (mmol/L)
   5) Glycemia (mmol/L)
   6) Serum insulin (U/l)

5. **Food consumption patterns:**

   1) **How many meals/days?**
      1) 1 meal
      2) 2 to 3 meals
      3) ≥4 meals
      4) More

2) **Number of fried foods away from home or Fast food/week**:
    1) Less than once/week
    2) 1 to 3 times per week
    3) 4-6 times per week
    4) Daily

3) **Raw or cooked vegetables (serving/day):**
    1) Less than 2 servings
    2) 2-3 servings
    3) More than 3 servings

4) **How often do you eat food that is fried at home?**
    1) Less than once/week
    2) 1-3 times per week
    3) 4-6 times per week
    4) Daily

5) **Kind of fat used for cooking or baking**:
    1) Butter
    2) Margarine
    3) Vegetable oil
    4) Two of them
    5) Three of them

6) What kind of oil is usually used for frying or baking at home (Sunflower oil, olive oil, Canola oil, Soya oil, etc.?

7) What kind of oil is usually used for salad dressings at home (Colza oil, olive oil, Canola oil, nut oil, etc.?

8) **Kind of milk or dairy products consumed:**
    1) Whole
    2) 2% or 1%
    3) Skim milk or dairy products
    4) Others (soya milk, rice milk)

9) **Kind of meat consumed:**
    1) Pork
    2) Mutton
    3) Veal
    4) Chicken
    5) Fish
    6) Seafood
    7) Goat

10) **Kind of bread consumed:**
    1) Refined wheat bread
    2) Whole wheat bread
    3) Others

11) **Kind of sugar consumed:**
    1) Table sugar
    2) Sweeteners
    3) No sugar

12) **How many teaspoons of sugar do you add to your beverages or food/day**? **…**
    1) None
    2) 1-2 teaspoons
    3) 3-4
    4) 5 or more

13) **How often do you consume chocolate, candy bars, molasses, jams and jellies, syrup, honey/week**?

    1) Never
    2) 1 to 2 times/week
    3) 3-5 times/week
    4) More

6. **Did you smoke cigarettes, cigars, pipes, water pipes, cigarillos in the last six months?**
    1) Yes
    2) No

7. **If Yes, how many cigarettes or cigars /day** (in case of cigarettes or cigars smoking):
    1) 1-4
    2) 5-14
    3) 15-24
    4) 25-34
    5) 35-44
    6) 45+

8. **If Yes, how many water pipes /week (**in case of water pipes smoking**):**
    1) 1 per week
    2) 2-3 per week
    3) >3 per week

9. Are you exposed to smoke more than 4 hours per day?

1) Yes
2) No

10. Physical activity: 1. Yes 2. No

Kind and frequency of physical activity:

1) Walking at a moderate pace 1. Yes 2. No
2) How often/week/day ………………
3) Jogging or running 1. Yes 2. No
4) How often/week/day ………………
5) Swimming, bicycling, tennis, aerobic dance 1. Yes 2. No
6) How often/week/day ………………
7) Body building, boxing, football game, ski or stair machine 1. Yes 2. No
8) How often/week/day ………………
9) Other vigorous activities (squash, lifting heavy objects) 1. Yes 2. No
10) How often/week/day ………………
11) Gardening or lawn mowing 1. Yes 2. No
12) How often/week/day ………………
13) Moderate activities such as general home exercise; pushing a vacuum cleaner, ironing, carrying groceries, climbing stairs) 1. Yes 2. No
14) How often/week/day
15) Other: 1. Yes 2. No
16) How often/week/day ……………….

11. Have you had any of this clinician -diagnosed illnesses?

1) Diabetes mellitus (type 2)
2) Cardiovascular disease such as Myocardial infarction, coronary bypass, stroke
3) Hypertension
4) 1+2
5) 1+2+3
6) 2+3
7) Others

12. Are you taking any regular medication? 1. Yes 2. No

If yes, which of the following are you taking?

1) Cholesterol lowering drugs (Statin)
2) Triglycerides lowering drugs
3) Hypoglycemic drugs
4) Anti-hypertensive drugs
5) Female hormones

6) Others

13. Days/week

1) 1
2) 2-3
3) 4-5
4) 6+

14. Do you currently take multi-vitamins? 1. Yes 2. No

If yes, how many tablets/per week (for the last six months)?

1) 2 or less
2) 3-5
3) 6-9
4) 10 or more

15. If you are currently taking them, what brand do you usually use and the exact dose/day (Specify)? ....

16. Do you currently take supplements? (Such as fish oil or others) 1. Yes 2. No

If yes, how many tablets/per weeks (for the last six months)

1) 2 or less
2) 3-5
3) 6-9
4) 10 or more

17. If you are currently taking them, what brand do you usually use and the exact dose/day (Specify)? ....

18. Do you ever follow a specific diet? 1. Yes 2. No

If yes, what type of diet?

1) High protein diet
2) Others

19. Family history of disease

1) 1. Yes   2. No
2) If yes, tick the appropriate box

	**Excess in weight/ Obesity**	**Type 2 Diabetes**	**Dyslipidemia or Hypertension or both**	**CVD**	**Fatty liver**
Father					
Mother					
Brother/s					
Sister/s					
Daughter/s					

Supplemental File 1
Questionnaire of the study: ***Relation of dietary patterns and nutritional profile to hepatic fibrosis in a sample of Lebanese NAFLD patients***

Food Frequency Questionnaire
*For each food listed, indicate how often on average you have used the amount specified during the past year

Identification number…………

		Average use last year								
**Daily products**		**Never or less than once per month**	**1-3 per month**	**1 per week**	**2-4 per week**	**5-6 per week**	**1 per day**	**2-3 per day**	**4-5 per day**	**6 + per day**
Milk (240ml)	Skim milk									
	Whole milk									
	1 or 2% milk									
	Soy milk									
Cream, non-dairy coffee whitener (exclude fat free) (1 tablespoon)										
Yogurt (113-170g)	Whole									
	Skim									
	Sweetened									
Cottage cheese [halloum,feta,mozarella (45g)]	Whole									
	Skim									
	1 or 2%									
French cheese (45g) or other cheese (Cheddar, American, etc.,)										
Labneh (45-50g)	Whole									
	Skim									
	1 or 2%									

**Fruits**	**Never or less than once per month**	**1-3 per month**	**1 per week**	**2-4 per week**	**5-6 per week**	**1 per day**	**2-3 per day**	**4-5 per day**	**6 + per day**
Bananas (1)									
Raisins or grapes (1/2 cup)									
Apples or pears (1)									
Prunes or dried plums (¼ cup or 6 dried)									
Oranges (1)									
Strawberries, fresh (1/2 cup)									
Peaches (1)									
Figs (2)									
Grapefruit (1/2) or grapefruit juices (small glass)									
Apricots (1 fresh, ½ cup canned or 5 dried)									
Orange juice (small glass) or other fruit juices									
Dates (Mejdool) (2)									
Avocado (1/2 fruit)									
Cantaloupe (1/4 melon) or watermelon (400g)									
Cherry (A dozen)									
Raspberry (1 cup)									
Others									
**Vegetables**									
Tomatoes (2 slices)									
Tomato juice (small glass)									

Tomato sauce (small glass)									
Broccoli (1/2 cup)									
Cauliflower (1/2 cup)									
Cabbage (1/2 cup)									
Carrots raw ½ carrot) or cooked carrot (1/2 cup)									
Corn (1/2 cup)									
Onions as a garnish or in salad (1 slice) or cooked (1/2 cup)									
Peppers (1/4 small)									
Eggplant, zucchini or summer squash (1/2 cup)									
Spinach cooked (1/2 cup)									
Spinach raw (1 cup)									
Icebergs or leaf lettuce (1 serving)									
	**Never or less than once per month**	**1-3 per month**	**1 per week**	**2-4 per week**	**5-6 per week**	**1 per day**	**2-3 per day**	**4-5 per day**	**6 + per day**
**Eggs, meat, ETC.**									
Eggs (1)									
Beef steak or roast (113-170g)									
Chicken (85g) or chicken/turkey sandwich									
Hamburger (1 patty)									
Pork or ham (113-170g)									
Fish (85-141 g)									
Bacon (2 slices)									
Hot-dog (1)									

Tuna steak (85-141g), canned tuna (85-113g)									
Shrimp, lobster (85-141g)									
**Breads, cereals, starches**									
Cooked Cornflakes (1 cup) or cold breakfast cereal (1 serving)									
Whole bread/white bread (1 slice)									
Bagels or Muffins (1)									
Biscuits (1)									
Manakish (150g) or ftayer (25g)									
Brown or white rice (cooked) (1 cup)									
Spaghetti, noodles (cooked) (1 cup)									
Potatoes, baked or boiled (1 cup)									
Potato chips (30 g) or French fries (170g)									
2 slices pizza									
	**Never or less than once per month**	**1-3 per month**	**1 per week**	**2-4 per week**	**5-6 per week**	**1 per day**	**2-3 per day**	**4-5 per day**	**6 + per day**
**Beans**									
Peas (1/2 cup)									
Beans, lima beans, fresh (1/2 cup)									
Lentils, fresh (1/2 cup)									
Chickpeas, fresh (1/2 cup)									
**Beverages**									
Coke, carbonated beverage (1 can)									
Sugar- free beverage (1 can) or 1 bottle									
Sport drink (1 can)									

Beer, regular (1 can or 1 bottle)									
Red/white wine (140g)									
Liquor, e.g., vodka, gin, etc. (un shot or 1 drink)									
Tea or coffee (including decaffeinated one) (226g, 1 cup)									
Dairy coffee drink (hot/cold), e.g., Cappuccino (453g)									
Plain water: bottled, sparkling or tap (227g)									
**Sweets, baked goods, Miscellaneous**	**Never or less than once per month**	**1-3 per month**	**1 per week**	**2-4 per week**	**5-6 per week**	**1 per day**	**2-3 per day**	**4-5 per day**	**6 + per day**
Milk chocolate (30 g)									
Dark chocolate (30 g)									
Doughnuts (1)									
Cake or pie, homemade or ready-made or Arabic pastries (1)									
Jams, honey, jellies, syrup, halawa, molasses (1 Tbs)									
Ketchup or red chili sauce (1 Tbs), tomato soup (1 cup)									
Peanuts, walnuts or other nuts (30g)									
Vegetable oil (1 Tbs), olives (10-16)									
Garlic, fresh or powdered (1 clove or 4 shakes)									
Mayonnaise or Mustard (1 Tbs)									
Tahini (1 Tbs)									
Energy or high protein Bars (1), snack bars (1)									
Popcorn (2-3 cups)									

Supplemental File 1

Questionnaire of the study: Relation of dietary patterns and nutritional profile to hepatic fibrosis in a sample of Lebanese NAFLD patients

dd/mm/yy

Questionnaire of the study: Relation of dietary patterns and nutritional profile to hepatic fibrosis in a sample of Lebanese NAFLD patients
(Exclusion Criteria)

These questions are designed to be filled or to be ticked according to the appropriate answer

Identification number……… **Phone Number…………….**

6. **Socio-demographic variables**

12) Nationality………
13) Place of residency………..
14) Place of birth…...
15) Age: ……….
16) Gender 1. Female 2. Male

7. **For each alcoholic beverage, tick the box indicating how often on average you have used the amount specified during the past year**

	**Never or less than once per month**	**1-3 per month**	**1 per week**	**2-4 per week**	**5-6 per week**	**1 per day**	**2-3 per day**	**4-5 per day**	**6 + per day**
**Beverages**									
Aperitif with alcohol									
Arak (1 glass of arak)									
Light beer (1 glass, bottle, can)									
Beer, regular (1 glass, 1 can or 1 bottle)									
Red/white wine (140g)									
Liquor, e.g., vodka, gin, whisky, cognac etc. (one shot or 1 drink)									

8. **Blood tests** (*exclusion criteria*)

HCV Ab………………

HBs Ag ………………….

HEV Ab………………….

Ac HBc Total…………….

AMA (Anti-Mitochondrial antibody) ………………….

α 1 anti trypsine (g/l)............ .

ANA (antinuclear antibody) ……………

Anti LKM (liver kidney microsome) ………………….

Ceruloplasmin (g/l) ………………………

Ferritin (ng/ml) ………………………

Transferrin saturation (%) ………………

9. **Did you take any of these medications the last six months**? 1. yes 2. No

If yes, did you take?

1) Amiodarone 1. yes 2. No
2) How often/week/day ………………
3) Antiretroviral drugs 1. yes 2. No
4) How often/week/day ………………
5) Aspirine 1. yes 2. No
6) How often/week/day ………………
7) Corticosteroïds 1. yes 2. No
8) How often/week/day ………………
9) Methotrexate 1. yes 2. No
10) How often/week/day ………………
11) Tamoxifen 1. yes 2. No
12) How often/week/day ………………
13) IV Tetracycline 1. yes 2. No
14) How often/week/day ………………
15) Synthetic estrogens 1. yes 2. No
16) How often/week/day
17) Others 1. yes 2. No
18) How often/week/day

10. Day/week

5) 1
6) 2-3
7) 4-5
8) 6+

11. Have you had any of these clinician -diagnosed illnesses?

1) Diabetes type 1
2) Ulcerative colitis/Crohn's
3) Gall bladder stones or any biliary diseases
4) Genetic metabolic disease
5) Auto-immune liver diseases
6) Recognized cirrhosis
7) Infection with Hepatitis A, B or C
8) Enteral or parenteral nutrition
9) Banding or jejunoileal bypass surgery
10) Polycystic ovary (female)

7. **Are you pregnant** (*female*)**?** 1. yes 2. No
