# Supplementary material for: Relation of Dietary Patterns and Nutritional Profile to Hepatic Fibrosis in a Sample of Lebanese Non-Alcoholic Fatty Liver Disease Patients
Source: Nutrients. 2022 Jun 20;14(12):2554. doi: 10.3390/nu14122554 (PMC9229197; doi:10.3390/nu14122554)
Supplement: Supplementary file 1 [file nutrients-14-02554-s001.zip › File S2.pdf]

Food Group	Included Food
**Vegetables**	Tomatoes, tomato juice, tomato sauce, broccoli, cauliflower, cabbage, carrots raw or cooked, corn, onions as a garnish or in salad or cooked, peppers, eggplant, zucchini or summer squash, spinach cooked or raw, icebergs or leaf lettuce
**Chickpeas, red beans, lentils, peas**	Fresh chickpeas, red beans, fresh lentils, peas
**Fruits and fruit juices**	Bananas, raisins, grapes, apples, pears, prunes, dried plums, oranges, fresh strawberries, peaches, figs, grapefruit, grapefruit juices, apricots, orange juice or other fruit juices, dates (Mejdool), avocado, cantaloupe, watermelon, cherry, raspberry
**Vegetable oil/olives**	Vegetable oil, tahini, olives
**Fish and sea food**	Fish, tuna steak, canned tuna, shrimps, lobster
**Almonds, walnuts, hazelnuts, sesames**	Almonds, walnuts, or other nuts
**Desserts, arabic pastries**	Dark chocolate, doughnuts, cake or pie (homemade or ready-made), Arabic pastries
**Beef meat**	Beef steak or roast
**Hamburger**	Hamburger
**Fries**	fries
**Pork**	Pork, bacon
**Pizza**	Pizza
**Spaghetti or noodles or cooked rice**	Spaghetti or noodles, cooked brown or white rice
**Chicken**	Chicken or turkey sandwich
**Carbonated beverages**	Coke, carbonated beverage
**Pies, fatayer**	Pies, fatayer (kind of pies)
**1 chicken egg**	1 chicken egg
**Fresh cream**	Cream, non-dairy coffee whitener
**Mayonnaise or mustard**	Mayonnaise or mustard
**Ketchup**	Ketchup
**Chips**	Chips
**Hot dog**	Hot dog
**Energy drink**	Energy drink
**Ham**	Ham
**Milk chocolate**	Milk chocolate
