# Supplementary material for: Relation of Dietary Patterns and Nutritional Profile to Hepatic Fibrosis in a Sample of Lebanese Non-Alcoholic Fatty Liver Disease Patients
Source: Nutrients. 2022 Jun 20;14(12):2554. doi: 10.3390/nu14122554 (PMC9229197; doi:10.3390/nu14122554)
Supplement: Supplementary file 1 [file nutrients-14-02554-s001.zip › File S3.pdf]

BMI; Body Mass Index

CDC; Centre for Disease Control

CDVD; Cardiovascular Disease

DHA; Docosahexaenoic Acid

DNL; De Novo Lipogenesis

ECLIA; Electrochemiluminescence Immunoassay

EPA; Eicosapentaenoic Acid

FFQ; Food Frequency Questionnaire

HOMA-IR; Homeostasis Model Assessment of Insulin Resistance

IDF; International Diabetes Federation

MUFA; Monounsaturated Fatty Acid

NAFLD; Non-alcoholic Fatty Liver Disease

NASH; Non-Alcoholic Steatohepatitis

NFS; NAFLD Fibrosis Score

ORs; Odd Ratios

PUFAs; Polyunsaturated Fatty Acids

USDA; US Department of Agriculture
